# Understanding the contrasting spatial haplotype patterns of malaria-protective β-globin polymorphisms

**DOI:** 10.1016/j.meegid.2015.09.018

**Published:** 2015-12

**Authors:** Carinna Hockham, Frédéric B. Piel, Sunetra Gupta, Bridget S. Penman

**Affiliations:** Department of Zoology, University of Oxford, Oxford, UK

**Keywords:** Sickle-cell, β-Thalassaemia, Malaria, Haplotype distribution, Gene conversion

## Abstract

The malaria-protective β-globin polymorphisms, sickle-cell (β^S^) and β^0^-thalassaemia, are canonical examples of human adaptation to infectious disease. Occurring on distinct genetic backgrounds, they vary markedly in their patterns of linked genetic variation at the population level, suggesting different evolutionary histories. β^S^ is associated with five classical restriction fragment length polymorphism haplotypes that exhibit remarkable specificity in their geographical distributions; by contrast, β^0^-thalassaemia mutations are found on haplotypes whose distributions overlap considerably. Here, we explore why these two polymorphisms display contrasting spatial haplotypic distributions, despite having malaria as a common selective pressure. We present a meta-population genetic model, incorporating individual-based processes, which tracks the evolution of β-globin polymorphisms on different haplotypic backgrounds. Our simulations reveal that, depending on the rate of mutation, a large population size and/or high population growth rate are required for both the β^S^- and the β^0^-thalassaemia-like patterns. However, whilst the β^S^-like pattern is more likely when population subdivision is high, migration low and long-distance migration absent, the opposite is true for β^0^-thalassaemia. Including gene conversion has little effect on the overall probability of each pattern; however, when inter-haplotype fitness variation exists, gene conversion is more likely to have contributed to the diversity of haplotypes actually present in the population. Our findings highlight how the contrasting spatial haplotype patterns exhibited by β^S^ and β^0^-thalassaemia may provide important indications as to the evolution of these adaptive alleles and the demographic history of the populations in which they have evolved.

## Highlights

• We model the spread of malaria-protective globin mutations on distinct haplotypes.
• We explore the effects of demographic and selective processes on their distributions.
• Mutation rate determines which spatial pattern is likely to occur.
• The structure of a meta-population and its level of migration are also critical.
• Gene conversion's contribution to diversity depends on haplotypic fitness variance.

## 1 Introduction

The mutations responsible for sickle-cell disease and β^0^-thalassaemia represent two unequivocal examples of balanced polymorphisms in the human genome (Hedrick, 2011, Roberts and Williams, 2003, Taylor et al., 2012). Occurring at high frequencies in many populations indigenous to malaria-endemic regions, these variants are subject to balancing selection due to their protective effect against *Plasmodium falciparum* malaria in the heterozygous state (Allison, 1964, Hill et al., 1991, Piel et al., 2010, Rockett et al., 2014, Williams et al., 2005a). Homozygotes suffer severe blood disorders (sickle-cell anaemia and β^0^-thalassaemia major, respectively), which, without access to diagnosis and treatment, are often lethal in the first few years of life (Weatherall et al., 2006).

In population genetics theory, it is generally accepted that natural selection results in one of two population genetic outcomes: (i) a *hard selective sweep*, in which a single adaptive allele sweeps rapidly through a population, resulting in the predominance of a single haplotype associated with the adaptive allele in the population, and (ii) a *soft selective sweep*, whereby ancestral genetic variation around the adaptive site is partially preserved owing to multiple alleles at the site being selected (Messer and Petrov, 2013, Ralph and Coop, 2010). In the context of this study, we define a haplotype as a set of DNA variations, including the variant under selection, that are located on a single chromosome and, by virtue of their close proximity, are inherited together. Both the sickle-cell mutation (β^S^) and β^0^-thalassaemia appear at first glance to be examples of soft selective sweeps. The former, which always results from the replacement of glutamic acid by valine at position 6 of the β-globin gene (*HBB* c. 20 A → T; p. Glu6-Val), is associated with five “classical” restriction fragment length polymorphism (RFLP) haplotypes (Table 1) (Flint et al., 1998). The latter results from any mutation that completely eliminates the production of protein from the β-globin gene (Weatherall and Clegg, 2001a). One-hundred and fifty-eight such mutations are currently reported (http://www.globin.bx.psu.edu/cgi-bin/hbvar/query_vars3, accessed 29 June 2015; Patrinos et al., 2004), and many of these can be found on more than one genetic background (Table 2) (Trabuchet et al., 1991, Weatherall and Clegg, 2001a).

The precise spatial patterns exhibited by β^S^- and β^0^-thalassaemia haplotypes are markedly different. For β^S^, current data suggests that the five classical haplotypes predominantly occupy geographically separate regions within Sub-Saharan Africa, the Middle East and India (Table 1) (Bitoungui et al., 2015, Flint et al., 1998, Gabriel and Przybylski, 2010, Hanchard et al., 2007). By contrast, whilst β^0^-thalassaemia mutations are mostly geographically specific on a cross-continental scale (Cao and Galanello, 2010, Weatherall and Clegg, 2001a) multiple variants can be found in the Mediterranean, the Middle East, and Asia, respectively, with their distributions considerably overlapping (Table 2). Furthermore, for each β^0^-thalassaemia variant, various associated genetic backgrounds typically coexist in the population (Table 2). To illustrate, the β^0^-thalassaemia mutation IVS-I-1 G → A is found on haplotypes V and II in Sicily (Schilirò et al., 1995), and haplotypes I, III, V, IX and A in Algeria (Bennani et al., 1994, Rouabhi et al., 1988). Similarly, the cd39 C → T mutation is associated with haplotypes I and II in Sicily, Sardinia and Corsica (Falchi et al., 2005), haplotypes I, II and IX in mainland Italy, and haplotypes I, II and B in Algeria (Lemsaddek et al., 2004).

In the case of β^0^-thalassaemia, different causal mutations have clearly arisen independently, whilst the occurrence of identical mutations on separate haplotypes is generally ascribed to gene conversion (Chen et al., 2007). For β^S^, however, it is commonly believed that each of the five classical β^S^-associated haplotypes represents five independent occurrences of the same A → T mutation in codon 6 of β-globin (Chebloune et al., 1988, Kulozik et al., 1986, Pagnier et al., 1984, Trabuchet et al., 1991, Wainscoat et al., 1983). Yet, as suggested by Livingstone in the 1980s, a single β^S^ mutation, and its subsequent transfer onto different haplotypic backgrounds by gene conversion, could also have generated the same present-day β^S^ pattern (Flint et al., 1998, Livingstone, 1989a, Livingstone, 1989b).

The different patterns exhibited by β^0^-thalassaemia and β^S^ mutations thus offer a unique opportunity to make a direct comparison between different sub-types of soft selective sweep in humans. Here, we identify the demographic and genetic processes that are more likely to give rise to either a sickle-cell-like or a β^0^-thalassaemia-like spatial distribution of haplotypes. Within the context of our spatial framework, we also specifically address the role that recurrent mutation and gene conversion may have played in the evolution of these polymorphisms.

## 2 Methods

### 2.1 The model

We simulated a meta-population of *Ne* diploid individuals, divided into *d* demes of equal size and arranged in a network with a varying degree of randomness in its migration connection structure, controlled by parameter *c* (see Penman et al., 2012, Watts and Strogatz, 1998; and Supplementary material for further details). Every generation, *Ne* increases by a percentage drawn from a uniform distribution between zero and a maximum possible population growth rate of *g*% (see Supplementary material). Any increase in total population size is spread equally across all demes.

The meta-population is initially monomorphic for the wild-type (β^A^) allele. In every generation *t*, within each deme, a finite set of potential offspring reaching reproductive age is generated according to (i) allele frequencies in that deme in generation *t-1*, (ii) mutation and/or gene conversion events (mutation rate = *μ* events per chromosome per generation; gene conversion rate = *r* events per chromosome per generation), and (iii) the relative fitness of each genotype, consistent with a standard model of the behaviour of a variant under selection. Following the generation of this offspring pool, individuals are randomly sampled without replacement to create the next generation of $\frac{Ne}{d}$ reproductively active adults in that deme. This step introduces the possibility of genetic drift. The final event to take place per generation is inter-demic gene flow. Each gene flow event involves the migration of *m*% of a deme's population to one of its linked partner demes in the migration network, and *vice versa* (further details in the Supplementary material).

### 2.2 Key model features/assumptions

#### 2.2.1 Mutation and gene conversion

We are only interested in the haplotypic diversity of β^S^- or β^0^-thalassaemia-bearing chromosomes. The model thus records only (i) mutation in the β^A^ → β^X^ direction, and (ii) the transfer of a β^X^ allele onto a new haplotypic background by gene conversion in β^A^β^X^ heterozygotes. All mutation and gene conversion rates throughout the manuscript refer to the rates at which these particular processes happen.

Every time a new β^X^ mutation arises, or an existing β^X^ mutation undergoes gene conversion, the resulting allele is assigned a unique numerical identifier representing a novel haplotypic background. This approach assumes a high diversity of pre-existing β-globin haplotypic backgrounds, such that each time a rare mutational or gene conversion event occurs it involves a different genetic background to any of those of previous mutations/gene conversions.

It is important to note that, given that the different haplotypes in our model can only arise through mutation or gene conversion (not reciprocal recombination), they are intended as proxies for β^S^- and β^0^-thalassaemia haplotypes whose occurrence cannot be accounted for by simple reciprocal recombination (*e.g.* the classical β^S^ haplotypes; Table 1) (Flint et al., 1998).

#### 2.2.2 Assignment of fitness values

Full details of the assignment of fitness values are provided in the Supplementary material. Crucially, the fittest individuals in our simulated populations were always β^A^β^X^ heterozygotes, who were assumed to experience malaria protection. Throughout all of our simulations, β^X^β^X^ homozygotes were assigned a fitness of zero and thus were not represented in the potential offspring pool.

It is possible for the haplotypic background of mutations to affect the course of β^0^-thalassaemia major or sickle-cell anaemia (Loggetto, 2013, Weatherall and Clegg, 2001b). However, given the absence, historically, of any effective treatment for these disorders, we have assumed that all individuals homozygous for β^S^- or β^0^-thalassaemia are likely to have been at a considerable disadvantage relative to the wild-type, regardless of whether they possessed a mutation with an ameliorating haplotypic background. We do, however, address the possibility of inter-haplotype fitness variation in our simulations, by incorporating a range, *f*, of possible heterozygote fitnesses into our model.

### 2.3 Parameterisation and implementation

The model was parameterised using value ranges taken from the literature where available (see Supplementary material). Of particular note, we tested four different allelic mutation rates: (i) 10^− 8^ events per chromosome per generation, *i.e.* the average nucleotide substitution rate for the human genome (Ellegren et al., 2003, Xue et al., 2009), although this yielded very few instances of a soft selective sweep in our simulations; (ii) 10^− 7^ events per chromosome per generation, accounting for the possibility of a higher-than-average rate of mutation in the β-globin cluster (Nachman and Crowell, 2000), and (iii) 5 × 10^− 7^ events per chromosome per generation and (iv) 10^− 6^ events per chromosome per generation to reflect the fact that hundreds of different types of mutations can give rise to a β^0^-thalassaemia allele. Results from the latter three mutation rates are presented here. The ranges of values used for all other parameters are described in the Supplementary material.

All simulations shown were run for 500 generations. Assuming a generation time of 15–25 years for humans, this represents 7.5 to 12.5 thousand years of malaria selection, which is consistent with estimates for how long *P. falciparum* is likely to have been a significant cause of human mortality (Carter and Mendis, 2002). Adaptive alleles arose stochastically throughout each simulation. We chose to analyse a “snapshot” of the genetic variation in the meta-population at the 500 generation time point.

Simulations were implemented in Matlab R2012b and performed on a 1728 2.0 GHz cores super-computer part of the Advanced Research Computing (ARC) resources at the University of Oxford.

### 2.4 Classification of evolutionary outcomes

Based on the reported geographical distributions of β^S^ and β^0^-thalassaemia variants and their associated haplotypes (Table 1, Table 2), we defined a series of possible outcomes within our model (see Sections 2.4.1–2.4.3). In particular, we sought to distinguish a spatially tessellating, ‘patchwork’ β^S^-like pattern and an overlapping β^0^-thalassaemia-like pattern.

#### 2.4.1 β^0^-Thalassaemia-like, or overlapping, outcome (Fig. 1A)

For this outcome, at least two different β^X^-associated haplotypes must be present in the meta-population. In addition, there must be sufficient overlap in the distributions of the different haplotypes that no more than 20% of demes contain a β^X^-associated haplotype that accounts for ≥ 95% of the haplotypic variation in the deme. We refer to such haplotypes henceforth as “dominating” haplotypes.

When assessing the geographical patterns exhibited by β^0^-thalassaemia (Table 2), we counted two different β^0^-thalassaemia mutations occurring on the same genetic background as two different haplotypes: considering the β^0^-thalassaemia mutation itself to be part of the haplotypic diversity in the population.

#### 2.4.2 β^S^-type, or patchwork, outcome (Fig. 1B)

For this outcome, there must be at least two different dominating β^X^-associated haplotypes in the whole meta-population, and at least 50% of the demes must contain a dominating β^X^-associated haplotype.

#### 2.4.3 Other possible model outcomes

Other possible model outcomes include: (i) no malaria-protective variation at the β-globin locus; (ii) a hard selective sweep, whereby malaria-protective variation is associated with only a single haplotype in the meta-population; (iii) the co-occurrence of malaria-protective variation on multiple haplotypes in the meta-population where haplotypes are completely deme-specific; and (iv) the co-occurrence of multiple haplotypes in the meta-population whose distributions are not deme-specific but do not reflect closely enough the overlapping (β^0^-thalassaemia) or patchwork (β^S^) spatial patterns defined above.

## 3 Results

### 3.1 Different mutation rates favour β^S^- or β^0^-thalassaemia-like patterns, subject to population size and structure

β^0^-Thalassaemic mutations can result from deletions, insertions and point mutations anywhere in the coding or regulatory region of β-globin. The β^S^ mutation, by contrast, is the result of the replacement of a specific, single nucleotide with another. There are therefore strong biological reasons to suppose that β^0^-thalassaemic mutations arise much more frequently than β^S^ mutations.

**Fig. 1:**
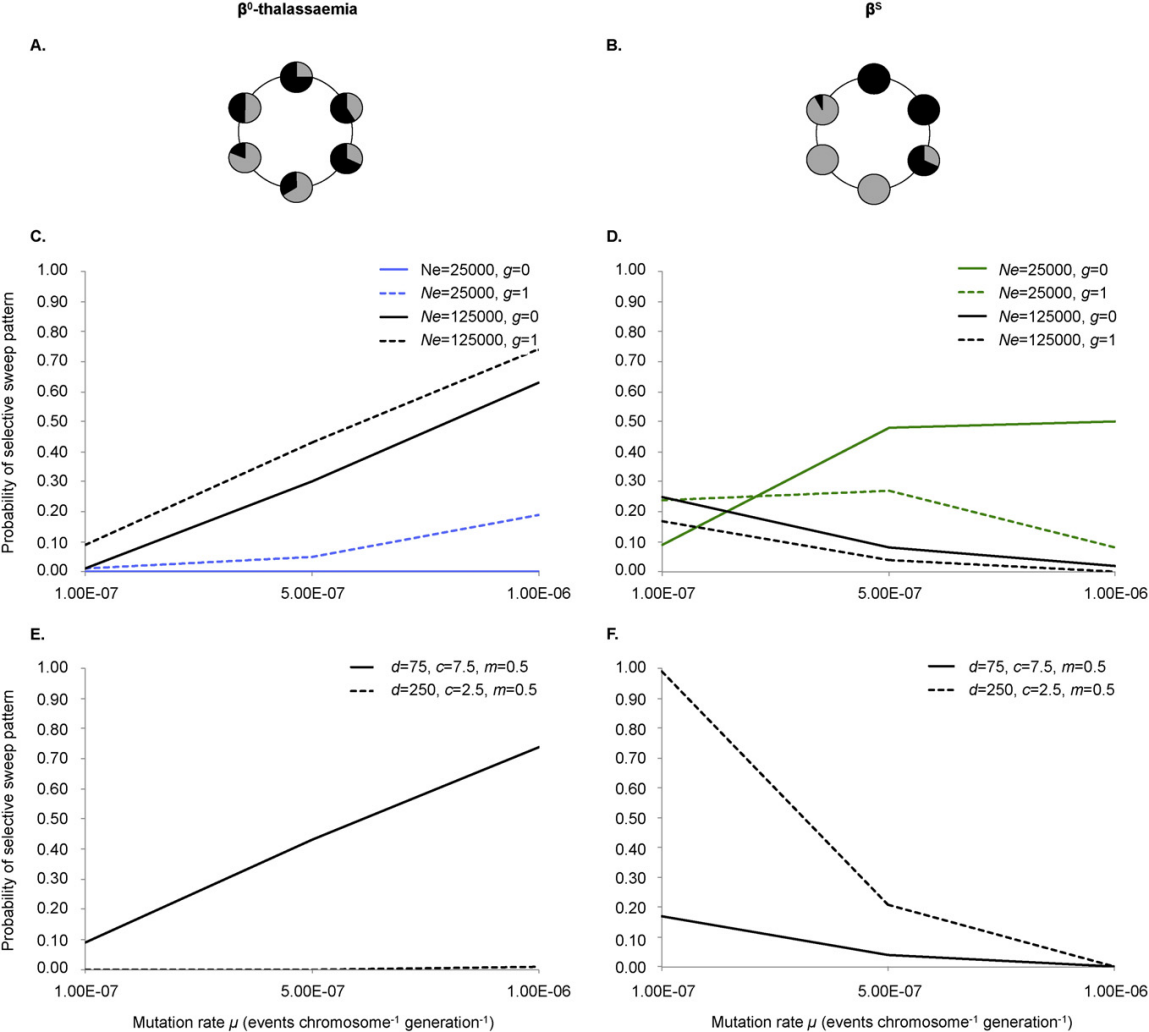
The β^0^-thalassaemia- and β^S^-like patterns and the effect of mutation rate on their likelihood of emergence. Panels (A) and (B) are abstract representations of the β^0^-thalassaemia- and β^S^-like outcomes of our model; each circle represents a deme, whilst different colours indicate whether one (one colour) or multiple (more than one colour) β^X^ haplotypes are present in a deme. In panels (C–F), each graph indicates how the probability of observing a β^0^-thalassaemia- (panels C and E) or β^S^-like (panels D and F) pattern changes with changing mutation rate (x-axis). Each data point is based on 100 simulations. In panels (C) and (D), the different lines represent different demographic scenarios relating to population size and growth rate. Other parameters were fixed as follows: *d* = 75, c = 7.5, *m* = 0.5, *f* = 0 and *r* = 10^− 6^. Increasing the mutation rate increases the probability of the β^0^-thalassaemia-like pattern, provided that initial total population size, *Ne,* and/or population growth rate, *g,* is sufficiently high. In panels (E) and (F), the different lines represent (i) a relatively weak population structure (fewer demes, more random connections: solid line), or (ii) a relatively strong population structure (many demes, fewer random connections: dashed line). Other parameter values were fixed as follows: *Ne* = 125,000, *G* = 1%, *f* = 0 and *r* = 10^− 6^. The effect of mutation rate on the probability of observing the β^0^-thalassaemia-like pattern is strongest for a weak population structure, whilst the opposite is true for the β^S^-like pattern.

In our simulations, increasing the mutation rate did increase the probability of observing the β^0^-thalassaemia-like pattern, but only if the overall population size was high (Fig. 1C). If the population was too small and/or too highly structured (*i.e.* with high population subdivision, a strong connection structure and low migration between demes), the probability of the β^0^-thalassaemia-like pattern remained low (< 0.20) even at the highest tested mutation rate. This was due to there being insufficient genetic variation or population movement to facilitate overlap in the haplotypes' distributions (Fig. 1C, E, respectively). The probability of a β^S^-like (patchwork) haplotype pattern, by contrast, was negatively correlated with mutation rate (Fig. 1D, F), except in the case of a low initial total population size (*Ne* = 25,000) and minimal population growth (*g* ≤ 0.5%) (Fig. 1D).

We also observed an interaction between the effects of initial total population size and population growth on the probability of the β^S^-like pattern at low mutation rates (Supplementary Fig. S1A). Population growth had a positive effect on the probability of the β^S^-like pattern when the initial total population size was small (*Ne* = 25000); such growth increased the overall size of the population, and thus the chances of more than one β^S^ haplotype arising anywhere. However, for a much larger starting population size (*Ne* = 125000) population growth had a slight negative effect. This is because increasing the size of an already large population led to too many β^S^ haplotypes arising through mutation and intermingling, thereby preventing a patchwork β^S^-like pattern. At a higher mutation rate (*μ* = 10^− 6^), population growth rate had a negative effect on the likelihood of the β^S^-like pattern across all initial population sizes.

### 3.2 A β^0^-thalassaemia-like pattern is most likely in a meta-population with a highly random connection structure, comprising few demes between which there is considerable gene flow; the opposite is true for a β^S^-like pattern

**Fig. 2:**
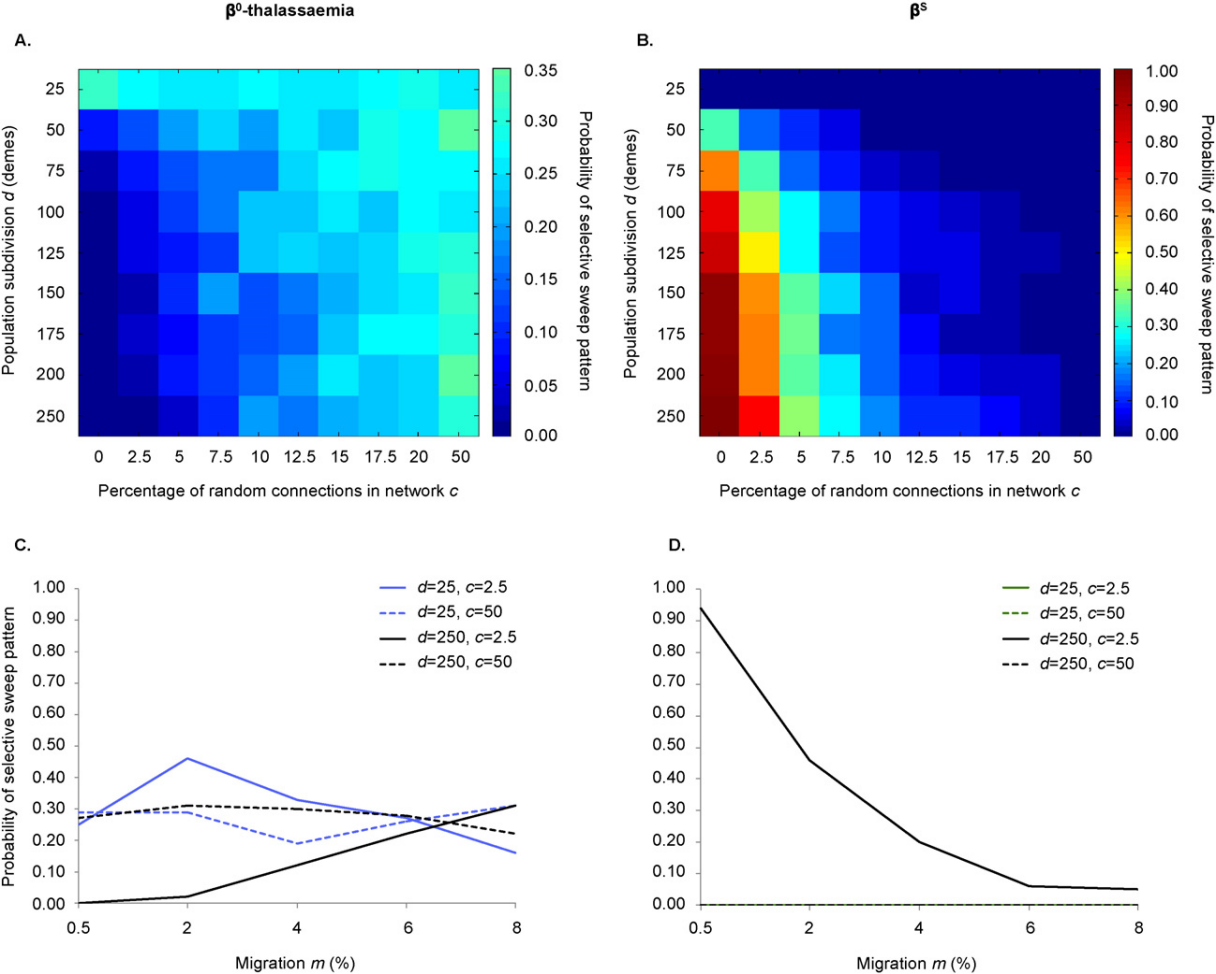
The combined effects of population subdivision, connection structure and migration on the probabilities of different types of selective sweep. The heatmaps indicate the effects of population subdivision and connection structure on the probability of observing β^0^-thalassaemia- (panel A) or β^S^-like (panel B) patterns. The colour of each square depicts the proportion of repeats (100 in total) that exhibit the pattern of interest. The x-axis of each panel indicates the percentage of connections in the network that are random, which corresponds directly to parameter *c* (see Supplementary material). Panels (C) and (D) indicate the additional impact of varying the level of gene flow (x-axis). Each data point is based on 100 simulations. Unless otherwise indicated in each panel, parameters were fixed as follows: *Ne* = 125,000, *g* = 1, *f* = 0, *r* = 0 and *μ* = 10^− 7^. In panels (A) and (B), results were averaged across two rates of migration: *m* = 0.5% and 2%. The β^0^-thalassaemia-like pattern is most likely when population subdivision is low and the connection structure is random; by contrast the β^S^-like pattern requires high population subdivision, a non-random connection structure and minimal gene flow to maximise its occurrence.

As illustrated in Fig. 2A and C, for a given initial total population size and rate of population growth, the β^0^-thalassaemia-like pattern was more likely under conditions of low subdivision, high migration and when the migration network contained more random connections. By contrast, the probability of obtaining the β^S^-like pattern was highest when population subdivision was high, the migration network was non-random and migration was low (Fig. 2B, D).

For both patterns of selective sweep, population subdivision and the degree of randomness in the migration network had a weaker effect at smaller initial population sizes when *μ* = 10^− 7^ events per chromosome per generation (Fig. S2, Fig. S3). Presumably this is because, even if the spread of alleles in the meta-population is slowed by high population subdivision or a highly non-random migration network, this has little impact if opportunities for new copies of the allele to arise are few. The converse is true for β^S^ when *μ* = 10^− 6^ events per chromosome per generation; in this case, the effects of population subdivision and connection structure are strongest when the initial total population size is small. Moreover, the effect of population subdivision was greatest when the connectivity network was non-random (Fig. 2A, B), indicating that even at high levels of subdivision a more random migration network is sufficient to allow the “small world” phenomenon (Watts and Strogatz, 1998, Watts, 1999) to occur, minimising the number of migratory steps that it takes for an allele to have access to the entire network.

### 3.3 When haplotype fitnesses vary, gene conversion is more likely to contribute to the haplotypic diversity of either β^0^-thalassaemia or β^S^

**Fig. 3:**
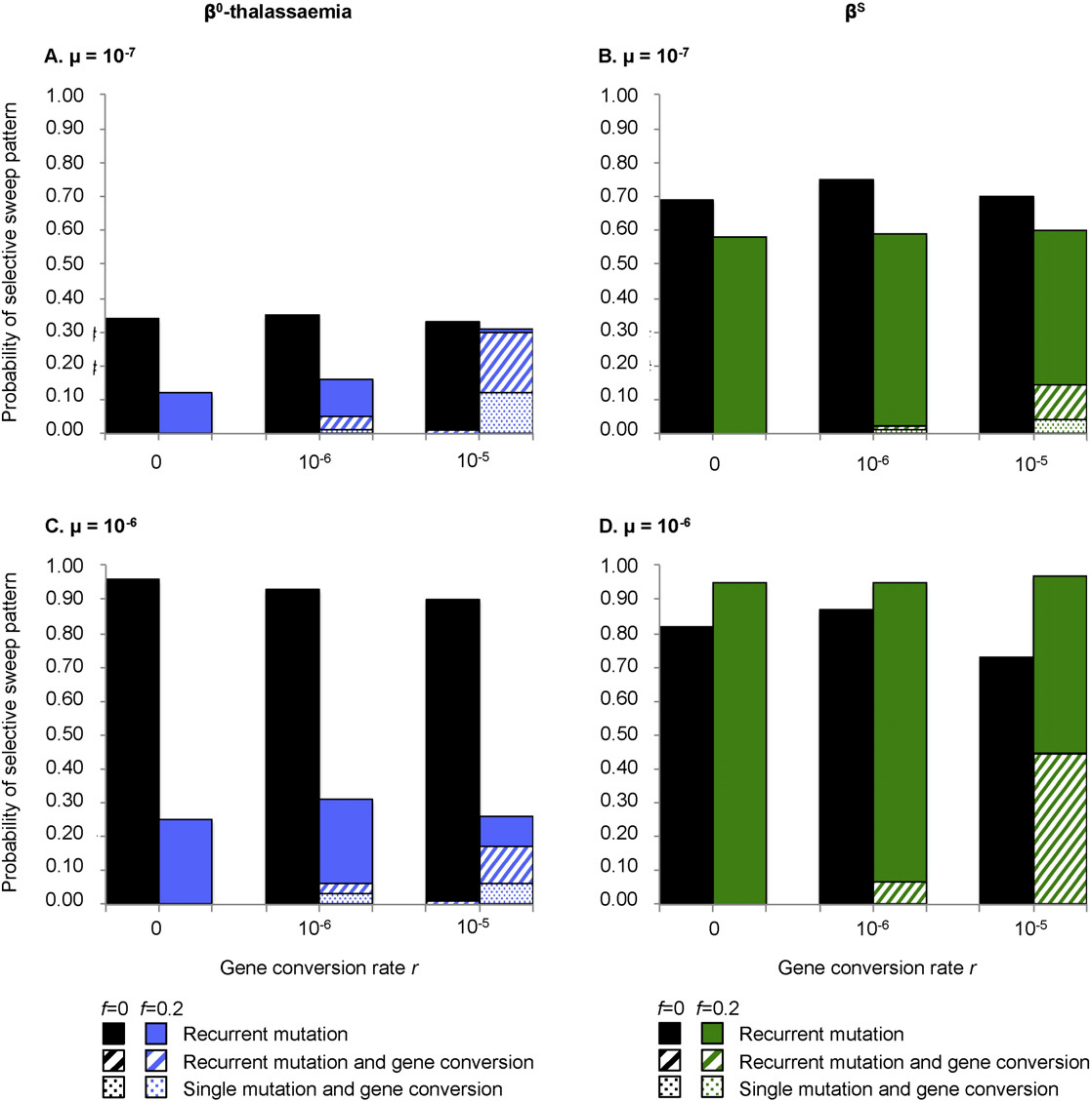
The effects of gene conversion and fitness variation on the probabilities of different types of selective sweep. The total height of each bar indicates the overall probability of observing a β^0^-thalassaemia-like (A,C) or β^S^-like (B,D) pattern at different levels of gene conversion (x-axis), and in the absence (black bars) or presence (blue or green bars) of fitness variation amongst heterozygotes. Each bar is based on 100 simulations, and is subdivided to indicate whether gene conversion and/or recurrent mutation was responsible for the haplotypic diversity present in the population at the end of each simulation (see legend to each graph). Results from two different mutation rates are shown: 10^− 7^ (A,B), and 10^− 6^ (C,D). Demographic parameter values in panels (A) and (C) were fixed as follows, to maximise the probability of a β^0^-thalassaemia-like scenario: *Ne* = 125,000, *g* = 1, *d* = 125, *c* = 15 and *m* = 2. Demographic parameter values in panels (B) and (D) were fixed as follows, to maximise the probability of a β^S^-like scenario: *Ne* = 25000, g = 1, *d* = 200, *c* = 2.5, *m* = 0.5. The inclusion of inter-haplotype fitness variation consistently decreases the probability of the β^0^-thalassaemia-like pattern. Whilst gene conversion has no effect on the overall probability of either patterns with or without inter-haplotype fitness variation, its relative contribution to the final haplotypic diversity increases with the rate of gene conversion if fitness variation is also present.

The β-globin cluster incorporates the γ- and δ-genes as well as an extensive locus control region (Forget and Hardison, 2009), mutations in any of which are capable of affecting the phenotypic outcome of a mutation in the coding region of β-globin itself. This phenomenon could be deemed epistasis, although the tight linkage between all elements of the β-globin cluster makes it equally acceptable to conceive of different haplotypes as allelic to one another. In either case, it is entirely plausible that β^S^- and β^0^-thalassaemia mutations could have different fitnesses according to their haplotypic background. We addressed the possibility of haplotypic fitness variation by randomly assigning a heterozygote fitness value, drawn from a predefined range of width *f*, to each haplotype as it arose (see Supplementary material). Including inter-haplotype fitness variation decreased the probability of observing the β^0^-thalassaemia-like pattern (Fig. 3A and C). This was also true for the β^S^-like pattern when *μ* = 10^− 7^ (Fig. 3B), although when *μ* = 10^− 6^, inter-haplotype fitness variation increased the probability of observing the β^S^-like pattern (Fig. 3D).

The rate of gene conversion had almost no effect on the overall probability of either the β^S^- or β^0^-thalassaemia-like pattern (Fig. 3). However, whenever gene conversion was allowed to occur, inter-haplotype fitness variation increased the proportion of scenarios in which haplotypes resulting from gene conversion formed part of the *final* haplotypic diversity of the population (Fig. 3). For example, for the β^0^-thalassaemia-like pattern, when the gene conversion rate was 10^− 5^ events per chromosome per generation and the mutation rate was 10^− 7^ events per chromosome per generation, gene conversion contributed to the final haplotypic diversity 97% of the time if *f* > 0, but < 1% of the time if *f* = 0 (Fig. 3A).

**Fig. 4:**
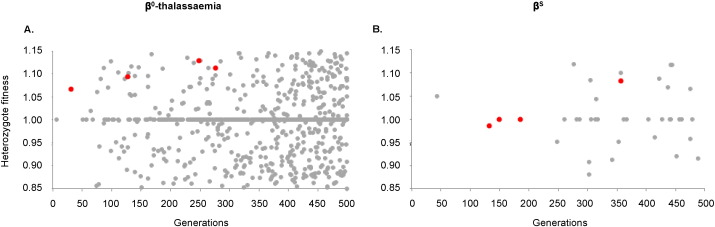
Comparing the fitnesses of all haplotypes that arose *versus* those that succeeded. Each panel indicates the heterozygote fitness values assigned to all haplotypes that arose over the course of a single simulation (grey), compared to those haplotypes that remained in the meta-population at a frequency ≥ 1% after 500 generations (red). The range of fitness values for the latter is considerably smaller than that for the former. Panel (A) illustrates a β^0^-thalassaemia-like scenario, for which the parameter values were: *Ne* = 125,000, g = 1, *d* = 125, *c* = 15, *m* = 2, *f* = 0.3, *r* = 10^− 5^ and *μ* = 10^− 7^. Panel (B) illustrates a β^S^-like scenario, for which the parameter values were *Ne* = 75,000, *g* = 0, *d* = 75, *c* = 5, *m* = 0.5, *f* = 0.3, *r* = 5 × 10^− 6^ and *μ* = 10^− 7^.

There has so far been no attempt to quantify the relative heterozygote fitnesses of different β^S^- and β^0^-thalassaemia-associated haplotypes, although clinical evidence does suggest that the severity of sickle-cell anaemia and β^0^-thalassaemia in homozygotes can vary according to haplotypic background (Ashley-Koch et al., 2000, Tsaras et al., 2009). It is therefore difficult to know whether the maximum fitness range included in Fig. 3 (*f* = 0.2) is plausible. Our model additionally shows that the haplotypes that coexisted in the long-term tended to be relatively similar in their heterozygote fitness, (Fig. 4), so a study carried out today may not provide a fair picture of what fitness variation could have existed in the past. Amongst *all* β^0^-thalassaemia-like results where *f* > 0, the average within-simulation heterozygote fitness range of haplotypes that coexisted in the meta-population after 500 generations was 0.03, compared to 0.18 for *all* haplotypes that arose during the 500 generations. For β^S^-like results, these values were 0.05 and 0.16, respectively. The average fitness range of dominating haplotypes was 0.01 for β^0^-thalassaemia-like repeats and 0.04 for β^S^-like repeats. The fittest haplotypes to arise contributed to the final haplotypic diversity in only 15% and 26% of all of the repeats exhibiting the β^S^- and β^0^-thalassaemia-like patterns, respectively, when *f* > 0.

## 4 Discussion

Across all of our simulations, once a deme had come to contain a β^S^- or β^0^-thalassaemia-associated haplotype at a high frequency, that deme was rarely taken over by another haplotype. This is because alleles that arose later contributed only a very small fraction of the adaptive variation in question and thus were vulnerable to loss by genetic drift. We refer to this phenomenon as “allelic exclusion” to coincide with the terminology used by Ralph and Coop (Ralph and Coop, 2010). Importantly, we found that the generation of either β^S^- or β^0^-thalassaemia-like patterns requires allelic exclusion to be undermined, but to different spatial extents; the β^S^-like pattern requires that allelic exclusion be maintained in parts of the meta-population but not the entire network, whilst the near complete avoidance of allelic exclusion is necessary for the β^0^-thalassaemia-like pattern.

Allelic exclusion can be avoided if a pre-existing haplotype has not yet reached a threshold frequency in a deme when subsequent haplotypes arrive. It follows that the timing of mutation, gene conversion and/or migration events within each deme is important in determining the evolutionary trajectory of the meta-population. Alternatively, allelic exclusion is undermined when genetic drift is weakened for incoming alleles, either through population growth or fitness variation between haplotypes. As illustrated in Fig. 1, Fig. 2, Fig. 3, the balance between all of these factors determines the probability of either a β^0^-thalassaemia- or β^S^-like pattern occurring. However, generally speaking, we expect the β^S^-like pattern to emerge when mutation rate is low and the population is highly subdivided, with low connectivity and little gene flow. The β^0^-thalassaemia-like pattern is more likely when mutation rate is high and the population is less subdivided, with high connectivity and high gene flow.

Several previous theoretical treatments of soft selective sweeps have delivered important insights into the genetic and demographic factors influencing the probability of adaptation by soft selective sweep *versus* hard selective sweep (see Messer and Petrov, 2013 for a full review). In a series of papers, Pennings and Hermisson used coalescent theory to show that soft sweeps are most likely when population size is large and/or allelic mutation rate is high (Hermisson and Pennings, 2005, Pennings and Hermisson, 2006). More recently, Ralph and Coop (2010) demonstrated that soft sweeps, specifically of the patchwork type, are likely to be common in species whose distributions are widespread and whose populations are geographically structured (Ralph and Coop, 2010). The behaviour of our model is consistent with these previous studies. By modelling a meta-population where we do not assume that different alleles exclude one another when they meet, we are also able to show that a mutation rate that is too high precludes the possibility of a β^S^-like soft selective sweep pattern, whilst a weaker geographical structure is important for the formation of a β^0^-thalassaemia-like pattern.

As noted in the Introduction, the occurrence of the same β^0^-thalassaemia variant on multiple haplotypes is generally attributed to gene conversion. Our results imply that gene conversion can contribute to haplotypic diversity only if inter-haplotype fitness is sufficiently variable. β^0^-Thalassaemia variants certainly vary in their clinical severity (Weatherall and Clegg, 2001a), often due to factors such as different levels of expression of foetal haemoglobin. No study has yet compared the relative level of malaria protection that is afforded by heterozygosity for different β^0^-thalassaemia haplotypes, but it is entirely possible that variable maintenance of foetal haemoglobin might affect malaria susceptibility (Amaratunga et al., 2011, Billig et al., 2012). Curiously, however, we found that, whilst including fitness variation made it more likely that gene conversion contributes to long-term haplotypic diversity, it simultaneously made the β^0^-thalassaemia-like pattern less likely (although not impossible). A specific combination of demographic conditions, gene conversion rate and inter-haplotype fitness variation, which increases the probability of observing a β^0^-thalassaemia-like pattern where the haplotypic diversity is partly derived from gene conversion, may yet be discovered.

Present consensus seems to be that gene conversion has had no role in the generation of the classical β^S^ haplotypes. This is despite modelling work by Livingstone in 1989, in which he used a stochastic model of the diffusion of different β^S^- and β^A^-associated chromosomes to demonstrate that reciprocal recombination and gene conversion readily give rise to multiple β^S^ haplotypes, with no need for recurrent mutation (Livingstone, 1989a). Our model demonstrates that a patchwork haplotype pattern that is at least partly derived from gene conversion is difficult to obtain unless inter-haplotype fitness variation exists. It is therefore possible that, until we understand what fitness variation is possible amongst β^S^ haplotypes, we will not be able to judge properly the role of gene conversion in its evolution. However, as indicated by our simulations, it is important to bear in mind that the observed present-day inter-haplotype fitness variation for both β^S^- and β^0^-thalassaemia may not necessarily reflect the full fitness range of all haplotypes that have arisen over the course of human evolutionary history and may not include the fittest haplotypes to have ever existed.

There is good evidence for past gene conversion events in the β-globin cluster (reviewed in Papadakis and Patrinos, 1999). Further and improved sequence data from this region of the genome will continue to provide insight into these processes, and may be able to indicate whether gene conversion has played a role in the generation of the classical β^S^ haplotypes. However, given that gene conversion events can involve a few hundred bases, which for a conversion event involving the β^S^ mutation is likely to include the highly conserved coding region of the β-globin gene, it may not always be possible to distinguish between *de novo* mutation and gene conversion at the β^S^ locus using sequence data alone.

We defined a patchwork β^S^-like pattern based on the geographical distribution of the classical β^S^ haplotypes. As noted in the Introduction, classical β^S^ haplotypes derive from RFLP analyses, which continue to be used in present-day studies of sickle-cell diversity (*e.g.* Bitoungui et al., 2015). Using SNP markers, Hanchard and colleagues showed that the classical Benin and Senegal haplotypes both exhibit a high degree of long-range haplotypic similarity extending across more than 400 kb in three separate populations (Hanchard et al., 2007). Similar results were found in a Ghanaian population (Ghansah et al., 2012). Fine-scale sequence analysis has revealed heterogeneities within the classical Benin and Bantu haplotypes (Bouhassira and Nagel, 1990, Patrinos et al., 2005). However, the distribution of the observed polymorphisms suggests that these differences evolved after the emergence of β^S^ on the distinct classical haplotypes, so as such, the broad pattern of the classical RFLP haplotypes remains.

β^0^-Thalassaemia mutations completely eliminate β-globin production from the affected gene. Other mutations exist which reduce but do not eliminate the production of β-globin, designated β^+^-thalassaemia alleles. Like β^0^-thalassaemia, β^+^-thalassaemia mutations are associated with multiple haplotypic backgrounds whose distributions have been found to overlap. To some extent, therefore, the results we present here for β^0^-thalassaemia also apply to β^+^-thalassaemia. However, our present assumption of zero fitness for homozygotes for the relevant mutation is less reasonable for certain milder β^+^-thalassaemic variants. We predict that allowing for milder homozygous, or compound heterozygous, phenotypes will allow an overlapping haplotypic pattern to be obtained over a still wider range of parameter space. We propose to explore this in the future as part of a model that allows a wider range of malaria-protective globin mutations to compete with one another.

Our work so far has focused on the β-globin locus. The study of the haplotypic evolution of α-globin will require a different modelling approach, incorporating duplicated α-globin genes in the α-globin cluster; the possible occurrence of the same variants in paralogous genes (Moradkhani et al., 2009); and the wide array of α-thalassaemia variants that are observed in human populations, including both single and double gene deletions (Weatherall and Clegg, 2001b). In this way, the relative contributions of recurrent mutation, gene conversion and unequal crossover in generating complex genetic variation in the α-globin gene cluster can be explored, along with the roles of malaria selection and demographic factors in shaping the spatial pattern of this diversity.

Our theoretical framework can be extended in a number of ways. One informative next step will be to allow β^S^ and β^0^-thalassaemia mutations to compete within the same interconnected network, alongside β^+^-thalassaemia mutations and other malaria-protective alleles with less severe clinical outcomes, for example HbC in Africa and HbE in Asia (Fucharoen and Weatherall, 2012, Piel et al., 2013). Further investigation into the origin, maintenance and fate of different β^0^-thalassaemia and β^S^ haplotypes will also need to consider the possible influence of epistasis between mutations at the α- and β-globin loci (Williams et al., 2005b, Penman et al., 2009, Penman et al., 2011); as well as interactions with other malaria-protective genetic variants elsewhere in the genome (reviewed in Hedrick, 2011, Kwiatkowski, 2005, Williams, 2006). Finally, Wilson and colleagues recently showed that, depending on their severity and frequency of recurrence, population bottlenecks can cause a soft selective sweep to become hard (Wilson et al., 2014). It would be interesting to see how their results relate to the specific case of β-globin polymorphisms under malaria selection.

## 5 Conclusion

Sickle-cell trait and β^0^-thalassaemia are two of our best examples of recent human evolution. Here we have shown that their differing selective sweep patterns may be just as much a product of different demographic conditions as they are of different mutation rates. Our results also suggest that inter-haplotypic fitness variation – a very real possibility for β-globin variants – both affects the probability of observing specific haplotype patterns and increases the probability of gene conversion having contributed to the variation present today. A better understanding of the fitness variation that is possible amongst β^0^-thalassaemia- or β^S^-associated haplotypes, will therefore be critical in determining the role of gene conversion in their evolution.

The following are the supplementary data related to this article.

Supplementary material: A detailed description of the model and its processes.

Fig. S1 The effects of population growth and initial total population size on the probability of the β^S^-like pattern. The total height of each bar indicates the overall probability of observing a β^S^-like pattern at different levels of maximum population growth rate (x-axis), and for different initial population sizes (see figure legend). Each bar is based on 100 simulations. The mutation rate was low (μ = 10^− 7^) in panel (A) and high (μ = 10^− 6^) in panel (B). Other parameter values are fixed as follows: *d* = 75, *c* = 7.5, *m* = 0.5, *f* = 0 and *r* = 10^− 6^. At the lower mutation rate, population growth rate increases the probability of the β^S^-like pattern when population size is small (*Ne* = 25,000) but decreases it when population size is large (*Ne* = 125,000). At a higher mutation rate, population growth has a consistent negative effect on the probability of the β^S^-like pattern.

Fig. S2 The interaction between the effects of population subdivision and initial total population size on selective sweep outcomes. Each graph indicates how the probability of observing a β^0^-thalassaemia-like (A,C) or β^S^-like (B,D) pattern changes with different levels of population subdivision (x-axis). Each data point is based on 100 simulations. Results are shown for two different initial population sizes: *Ne* = 25000 (solid line) and *Ne* = 125000 (dashed line). Two different mutation rates are also shown: *μ* = 10^− 7^ (A,B) and *μ* = 10^− 6^ (C,D). In panels (A) and (C), *m* = 2, to maximise the probability of a β^0^-thalassaemia-like pattern; in panels (B) and (D) *m* = 0.5, to maximise the probability of a β^S^-like pattern. Other parameter values are fixed as follows: *g* = 0, *c* = 7.5, *f* = 0 and *r* = 10^− 6^. When both population size and mutation rate are low, opportunities for new copies of the allele to arise are few and therefore the speed at which alleles move through the network is less important in determining the patterns that emerges. By contrast, when both population size and mutation rate are high, so many haplotypes are generated that the β^0^-thalassaemia-like pattern is guaranteed whilst the β^S^-like pattern is precluded; regardless of how easy or difficult it is for alleles to move through the network.

Fig. S3 The interaction between the effects of network connection structure and initial total population size on selective sweep outcomes. Each graph indicates how the probability of observing a β^0^-thalassaemia-like (A,C) or β^S^-like (B,D) pattern changes with different degrees of randomness in the migration network connection structure (x-axis). Each data point is based on 100 simulations. Results are shown for two different initial population sizes: *Ne* = 25,000 (solid line), and *Ne* = 125,000 (dashed line). Two different mutation rates are also shown: *μ* = 10^− 7^ (a,b) and *μ* = 10^− 6^ (c,d). In panels (A) and (C), *m* = 2, to maximise the probability of a β^0^-thalassaemia-like pattern; in panels (B) and (D) *m* = 0.5, to maximise the probability of a β^S^-like pattern. Other parameter values are fixed as follows: *g* = 0, *d* = 75, *f* = 0 and *r* = 10^− 6^. As for Supplementary Fig. S2, when both population size and mutation rate are low, opportunities for new copies of the allele to arise are few, and therefore the speed at which alleles move through the network is less important in determining the patterns that emerges. By contrast, when both population size and mutation rate are high, so many haplotypes are generated that that the β^0^-thalassaemia-like pattern is guaranteed whilst the β^S^-like pattern is precluded, regardless of how easy or difficult it is for alleles to move through the network.
The interaction between the effects of network connection structure and initial total population size on selective sweep outcomes. Each graph indicates how the probability of observing a β^0^-thalassaemia-like (A,C) or β^S^-like (B,D) pattern changes with different degrees of randomness in the migration network connection structure (x-axis). Each data point is based on 100 simulations. Results are shown for two different initial population sizes: *Ne* = 25,000 (solid line), and *Ne* = 125,000 (dashed line). Two different mutation rates are also shown: *μ* = 10^− 7^ (a,b) and *μ* = 10^− 6^ (c,d). In panels (A) and (C), *m* = 2, to maximise the probability of a β^0^-thalassaemia-like pattern; in panels (B) and (D) *m* = 0.5, to maximise the probability of a β^S^-like pattern. Other parameter values are fixed as follows: *g* = 0, *d* = 75, *f* = 0 and *r* = 10^− 6^. As for Supplementary Fig. S2, when both population size and mutation rate are low, opportunities for new copies of the allele to arise are few, and therefore the speed at which alleles move through the network is less important in determining the patterns that emerges. By contrast, when both population size and mutation rate are high, so many haplotypes are generated that that the β^0^-thalassaemia-like pattern is guaranteed whilst the β^S^-like pattern is precluded, regardless of how easy or difficult it is for alleles to move through the network.

## Figures and Tables

**Table 1:** Distribution of β^S^ haplotypes. Relative percentages (%) of classical β^S^-associated haplotypes in different geographical regions. Classical β^S^ haplotypes are defined as those which cannot be accounted for by reciprocal recombination. Countries where β^S^ has largely been imported, for example parts of Western Europe and North America, are not included as these do not reflect the early evolutionary history of the variant.

		β^S^-associated haplotypes						
Region	Country	Arab-Indian	Benin	Cameroon	Central African Republic	Senegal	Other	Reference
Sub-Saharan Africa	Angola	–	12.0	–	88.0	–	–	(Flint et al., 1998)
Benin	–	100.0	–	–	–	–	(Gabriel and Przybylski, 2010)
Burkina Faso	–	100.0	–	–	–	–	(Gabriel and Przybylski, 2010)
Cameroon	–	83.7	16.3	–	–	–	(Flint et al., 1998)
CAR	–	6.9	3.4	82.8	3.4	3.5	(Flint et al., 1998)
Kenya	–	1.3	–	98.2	–	0.5	(Flint et al., 1998)
Nigeria	–	92.9	3.4	0.7	0.9	2.1	(Flint et al., 1998)
Senegal	–	14.0	–	1.8	80.7	3.5	(Flint et al., 1998)
Tanzania	–	–	–	100.0	–	–	(Flint et al., 1998)
Togo	–	100.0	–	–	–	–	(Gabriel and Przybylski, 2010)
North Africa & Middle East	Algeria	–	100.0	–	–	–	–	(Flint et al., 1998)
Egypt	–	100.0	–	–	–	–	(Gabriel and Przybylski, 2010)
Morocco	–	100.0	–	–	–	–	(Flint et al., 1998)
Saudi Arabia	1.5	98.5	–	–	–	–	(el-Hazmi et al., 1999)
Saudi Arabia	94.0	–	–	4.0	–	2.0	(Kulozik et al., 1986)
Kuwait	77.8	16.7	–	–	–	5.5	(Adekile et al., 1994)
Bahrain	90.0	2.5	–	5.0	–	2.5	(Al Arrayed and Haites, 1995)
Syria	–	100.0	–	–	–	–	(Flint et al., 1998)
Tunisia	–	94.8	–	–	–	5.2	(Flint et al., 1998)
Turkey	0.4	96.3	–	–	–	3.3	(Flint et al., 1998)
Turkey	–	100.0	–	–	–	–	(Gabriel and Przybylski, 2010)
South Asia	India	100.0	–	–	–	–	–	(Gabriel and Przybylski, 2010)
India	90.7	–	–	–	–	9.3	(Labie et al., 1989)
India	98.45	–	1.55	–	–	–	(Oner et al., 1992)
India	91.67	–	2.78	1.39	–	4.17	(Niranjan et al., 1999)

**Table 2:** Distribution of β^0^-thalassaemia variants and their associated haplotypes. Relative percentages (%) of β^0^-thalassaemia variants and their associated haplotypes in a range of geographical settings. Haplotype definitions are given in Antonarakis et al. (1985).

Region	Country	β^0^-Thalassaemia variants and their associated haplotypes									Reference
North Africa & Middle East		Cd37 (G→A)	Cd39 (C→T)	IVS-I-1 (G→A)	IVS-I-2 (T→C)	IVS2-1 (G→A)	FSC-8 (-AA)	FSC-6 (-A)	IVS-I-2 (T→G)	Other	
	Algeria	–	44.59 (I, II)	18.91 (I, III, V, IX, A)	5.40	–	–	27.70 (I, IX, A)	–	3.38	(Bennani et al., 1994)
	Algeria	–	50.00	11.90	19.05	–	–	16.67	–	2.38	(Bouhass et al., 1994)
	Morocco	4.67 (I)	39.26 (I, II Nd)	7.48 (V, IX)	7.48 II, IX)	3.74 (III)	20.56 (IV, VI, VII)	8.41 (III, IX)	4.67 (IX)	3.74 (IX, III)	(Agouti et al., 2008)
	Morocco	3.51 (VII, I)	24.56 (1, II)	21.05 (IV, V, IX)	5.26 (II, IX)	1.75 (III)	24.56 (IV, VI)	15.79 (IX, III)		3.51 (IX)	(Lemsaddek et al., 2003)
	Morocco	1.55	37.98 (I, II)	12.40 (IV, V, IX)	3.10	–	13.95 (IV, VI, VII)	19.38 (III, IX, A)	0.78	10.85	(Lemsaddek et al., 2004)
	Tunisia	–	62.07	–	–	–	3.45	3.45	3.45	27.59	(Fattoum et al., 1991)

Mediterranean & Europe		Cd8 (-AA)	Cd39 (C→T)	IVSI-1 (G→A)	IVS-I-2 (T→A)	IVS2-1 (G→A)	Cd6 (-A)	–	–	Other	

	Albania	–	65.00	15.00	–	5.00	–	–	–	15.00	(Boletini et al., 1994)
	Greece	–	50.86	39.66	–	6.03	–	–	–	3.45	(Boletini et al., 1994)
	Macedonia	–	21.43	59.52	–	7.14	–	–	–	11.90	(Boletini et al., 1994)
	Sardinia	–	97.80 (1, 2, 3, 4, 5)	0.035	–	0.035	2.13 (IX)	–	–	–	(Cao et al., 1991)
	Sicily	–	79.35 (I, II)	20.65 (II, V)	–	–	–	–	–	–	(Schilirò et al., 1997)
	Sicily	–	71.92 (I, II)	18.72 (II, V)	3.45	3.94	1.23	–	–	0.74	(Schilirò et al., 1995)

Asia		Cd8/9 (+ G)	Cd15 (G→A)	Cd30 (G→C)	Cd41/42 (TCTT)	IVSI-1 (G→T)	IVSII-654 (C→T)	− 619 bp del	–	Other	

	India	6.25 (3 haps.)	2.08 (1 hap.)	2.08 (1 hap)	10.42 (3 haps.)	12.50 (2 haps.)	–	2.08 (1 hap.)	–	64.58	(Gupta et al., 2008)
	Maldives	–	–	60.00 (B, C)	5.00 (F)	–	–	–	–	0.35	(Furuumi et al., 1998)
