# Supplementary material for: Understanding the contrasting spatial haplotype patterns of malaria-protective β-globin polymorphisms
Source: Infect Genet Evol. 2015 Dec;36:174–83. doi: 10.1016/j.meegid.2015.09.018 (PMC4653953; doi:10.1016/j.meegid.2015.09.018)
Supplement: Supplementary file 1 — Supplementary material: A detailed description of the model and its processes. [file mmc1.doc]

# Supplementary material: A detailed description of the model and its processes

## As mentioned in *Methods*, our model follows a standard model of the behaviour of a variant under selection, and can be represented by the following equation:

$G_{het_{i}(t)}=\frac{2.p_{i(t)}(1-\sum_{j=1}^{n} p_{j(t)}).w_{het_{i}}}{(1-\sum_{j=1}^{n} p_{j(t)})^{2}.w_{AA}+\sum_{k=1}^{n} (2.p_{k(t)}(1-\sum_{j=1}^{n} p_{j(t)}).w_{het_{k}})}$ **(1)**

where $G_{het_{i}(t)}$ represents the frequency of β^A^β^X^_i_ heterozygotes in the deme’s offspring pool in generation *t*; $p_{i(t)}$ is the frequency of mutant haplotype β^X^_i_ in the deme in generation *t* following mutation and/or gene conversion; $w_{het_{i}}$ is the relative fitness of genotype β^A^β^X^_i_; $w_{AA}$ is the relative fitness of genotype β^A^β^A^; and *n* is the total number of β^X^ haplotypes present in the deme. In scenarios where $w_{het_{i}}$ is varied, the maximum range of possible heterozygote fitnesses ($w_{het_{i}(\max)}-w_{het_{i}(\min)}$) was *f*. Further details of the assignment of fitness values are provided below.

Sickle-cell anaemia and β^0^-thalassaemia are both lethal if untreated (Weatherall et al., 2006). Given the absence of effective treatment in early human populations, we have assumed the fitness of any β^X^ homozygote to be equal to 0, therefore:

$G_{AA(t)}=1-\sum_{j=1}^{n} G_{het_{j}(t)}$ **(2)**

where $G_{AA(t)}$ represents the frequency of β^A^β^A^ homozygotes in the deme in generation *t*.

## Population size and population growth

We tested different values of initial total population size between 25,000 and 125,000 in increments of 25,000, to reflect likely historic population sizes (Atkinson et al., 2009). Parameter *g*, the maximum possible population growth rate in any given simulation, was varied between 0% and 1%. This is based on reported estimates of historic human population growth (McEvedy and Jones, 1978).

## Population subdivision and connection network

The number of demes *d* in the meta-population was fixed for each simulation. We tested values of *d* between 25 and 250 in increments of 25-50. As in Penman et al. (2012), we used the method outlined by Watts and Strogatz (1998) to generate population networks demarcating the migratory connections (edges) between the constituent demes (nodes) (Penman et al., 2012; Watts and Strogatz, 1998). Briefly, the level of connectivity within a meta-population is determined by the percentage *c* of demes that are connected to a randomly chosen partner deme. When *c* = 0, each deme is connected to its two nearest neighbors in a ring-shaped network, and the average shortest path length between any two demes is at a maximum. As *c* increases, randomly chosen connections are re-wired and the average shortest path length between any two demes decreases. We varied the value of *c* between 0% and 50%, since preliminary simulations showed that higher *c* values yielded similar results to *c* = 50.

## Mutation and gene conversion

Different haplotypes in our model can only arise through mutation or gene conversion, not reciprocal recombination. They are therefore intended as proxies for haplotypes whose occurrence cannot be accounted for by simple recombination, for example the major β^S^ haplotypes.

The meta-population is initially monomorphic for the ancestral β^A^ allele. In every generation, within each deme, the number of mutant β^X^ alleles entering the deme is drawn from a Binomial distribution, where the number of “trials” equals the number of wild-type chromosomes in that deme and the probability of success is determined by the per chromosome mutation rate *μ*. Four different allelic mutation rates are explored: (i) 10^-8^ events per chromosome per generation, (ii) 10^-7^ events per chromosome per generation, (iii) 5x10^-7^ events per chromosome per generation, and (iv) 10^-6^ events per chromosome per generation. Only results from the latter three are presented, as the lowest mutation rate yielded very few instances of the β^S^- or β^0^-thalassaemia-like patterns.

If a β^X^-associated chromosome is present in a deme, the number of gene conversion events involving the transfer the β^X^_i_ mutation onto haplotypic background *i+1* is again drawn from a Binomial distribution. In this instance, the number of “trials” is the number of β^A^β^X^ heterozygotes in the deme and the probability of success is the per chromosome gene conversion rate *r*. Crucially, the number of “trials” equalling the number of β^A^β^X^ heterozygotes captures the fact that gene conversion can only occur within heterozygotes, making the deme-level gene conversion rate markedly lower than it would be if gene conversion occurred independently to an individual’s genotype. Given the lack of evidence for the per chromosome gene conversion rate at the β-globin locus, we varied this value between 0 events per chromosome per generation and the extreme value of 5x10^-5^ events per chromosome per generation.

Each β^X^_i_ allele is labeled according to its associated haplotypic background, with any mutation arising *de novo* or undergoing gene conversion being assigned a new haplotypic background. Although a simplification, this allowed individual mutant alleles to be tracked separately within the meta-population.

## Gene flow

During every iteration of each simulation, following reproduction and the ascertainment of successful offspring in each deme, *d* demes were chosen to undergo bi-directional gene flow with a randomly chosen partner deme to which they were directly linked in the migration network. These *d* demes were sampled with replacement from all of the demes present in the meta-population, thus a gene flow event may not occur in every deme in every generation or may, conversely, occur more than once per generation for some demes. Every gene flow event between two demes involves the exchange of a randomly chosen *m*% of the individuals in each of the partner demes. The value of *m* was varied between 0.5% and 8% in increments of 1.5-2%.

## Malaria selection and genotype fitness

Genotypes β^X^β^X^ were always assigned a fitness of 0, reflecting their historical lethality. As in Penman et al. (2009, 2012), we calculate genotype fitness values for β^A^β^A^ and β^A^β^X^ based on presumed mortality rates that incorporate mortality due to *falciparum* malaria. Each genotype was assigned a mortality rate *of* *q* + *γφ*, where *q* is the baseline mortality rate of the β^A^β^A^ and β^A^β^X^ genotypes in the absence of malaria, *γ* is the excess mortality due to malaria and *φ* is the relative risk of death from malaria for that genotype. The fitnesses of the genotypes are then calculated as the ratio of the average survival time of each genotype (the reciprocal of the total mortality rate of the genotype) to the maximum total survival time of any genotype in the population. A value of *q* of 0.04 years^-1^ is assigned to both β^A^β^A^ and β^A^β^X^ to reflect the absence of any pathology in β^A^β^X^ heterozygotes. The values of *γ* and *φ* are: *γ* = 0.008 years^-1^, *φ* = 1 for β^A^β^A^ and *φ* = 0.06 for β^A^β^X^.

When inter-haplotype fitness variation was absent, any β^A^β^X^_i_ heterozygote was assigned the maximum possible fitness in the population (*w_i_* always = 1). However, as mentioned previously, we also sought to address the possibility of variation in fitness between the heterozygotes carrying different β^S^ or β^0^-thalassamia haplotypes (Ashley-Koch et al., 2000; Tsaras et al., 2009). When this was allowed to occur, there was an equal probability that a specific heterozyote should be assigned a fitness of 1 (*w_i_* = 1) or that the fitness of a specific heterozygote should be drawn from a uniform distribution between 1 – *f*/2 and 1 + *f*/2 (1 – *f*/2<*w_i_*<1 + *f*/2). Thus, the majority of haplotypes had a heterozygote fitness of 1 and the total possible range of heterozygote fitnesses was *f*. Due to uncertainty in the degree of fitness variation for β^S^ and β^0^-thalassaemia, we varied *f* between 0 and 0.3 to allow for a wide spectrum of evolutionary scenarios.

# References for Supplementary Text

Ashley-Koch, A., Yang, Q., Olney, R.S., 2000. Sickle hemoglobin (HbS) allele and sickle cell disease: a HuGE review. Am. J. Epidemiol. 151, 839–45.

Atkinson, Q.D., Gray, R.D., Drummond, A.J., 2009. Bayesian coalescent inference of major human mitochondrial DNA haplogroup expansions in Africa. Proc. Biol. Sci. 276, 367–73. doi:10.1098/rspb.2008.0785

Ellegren, H., Smith, N.G., Webster, M.T., 2003. Mutation rate variation in the mammalian genome. Curr. Opin. Genet. Dev. 13, 562–568.

McEvedy, C., Jones, R., 1978. Atlas of world population history. Penguin.

Nachman, M.W., Crowell, S.L., 2000. Estimate of the Mutation Rate per Nucleotide in Humans. Genetics 156, 297–304.

Penman, B.S., Gupta, S., Buckee, C.O., 2012. The emergence and maintenance of sickle cell hotspots in the Mediterranean. Infect. Genet. Evol. 12, 1543–50.

Penman, B.S., Pybus, O.G., Weatherall, D.J., Gupta, S., 2009. Epistatic interactions between genetic disorders of hemoglobin can explain why the sickle-cell gene is uncommon in the Mediterranean. Proc. Natl. Acad. Sci. U. S. A. 106, 21242–6.

Tsaras, G., Owusu-Ansah, A., Boateng, F.O., Amoateng-Adjepong, Y., 2009. Complications associated with sickle cell trait: a brief narrative review. Am. J. Med. 122, 507–12. doi:10.1016/j.amjmed.2008.12.020

Watts, D.J., Strogatz, S.H., 1998. Collective dynamics of “small-world” networks. Nature 393, 440–2.

Weatherall, D., Akinyanju, O., Fucharoen, S., Olivieri, N., Musgrove, P., 2006. Inherited Disorders of Hemoglobin.

Xue, Y., Wang, Q., Long, Q., Ng, B.L., Swerdlow, H., Burton, J., Skuce, C., Taylor, R., Abdellah, Z., Zhao, Y., MacArthur, D.G., Quail, M.A., Carter, N.P., Yang, H., Tyler-Smith, C., 2009. Human Y chromosome base-substitution mutation rate measured by direct sequencing in a deep-rooting pedigree. Curr. Biol. 19, 1453–7.
