# Supplementary figures and images for: Understanding the contrasting spatial haplotype patterns of malaria-protective β-globin polymorphisms
Source: Infect Genet Evol. 2015 Dec;36:174–83. doi: 10.1016/j.meegid.2015.09.018 (PMC4653953; doi:10.1016/j.meegid.2015.09.018)

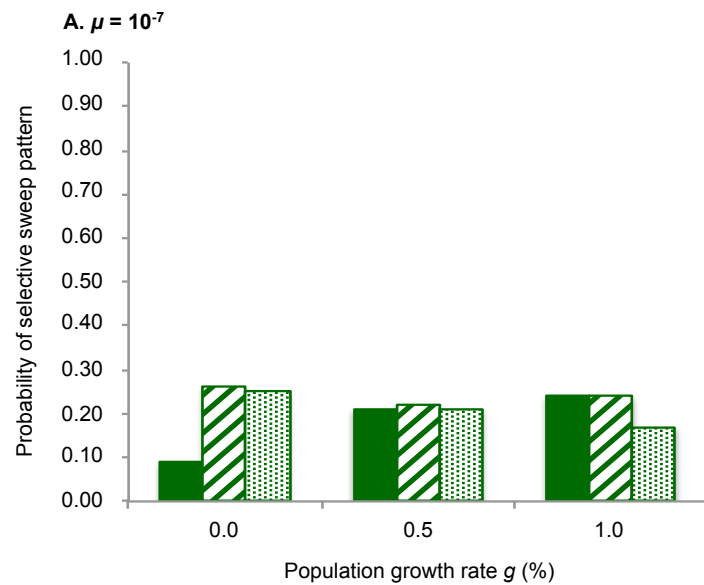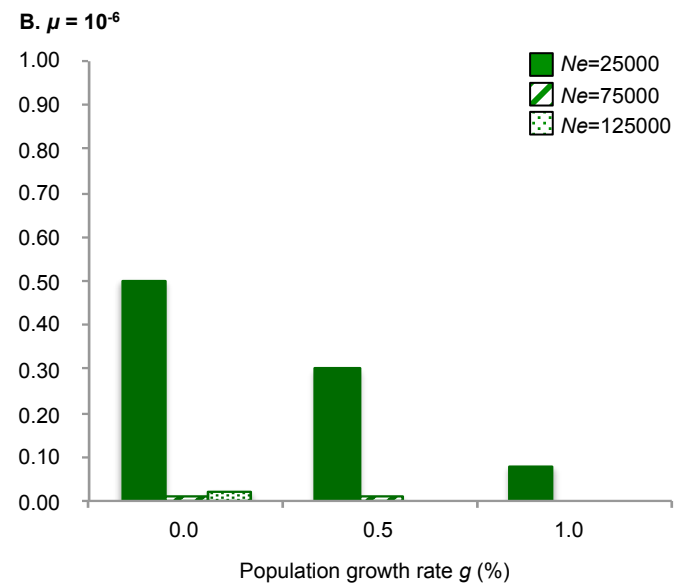

Supplement: Fig. S1 — The effects of population growth and initial total population size on the probability of the βS-like pattern. The total height of each bar indicates the overall probability of observing a βS-like pattern at different levels of maximum population growth rate (x-axis), and for different initial population sizes (see figure legend). Each bar is based on 100 simulations. The mutation rate was low (μ = 10− 7) in panel (A) and high (μ = 10− 6) in panel (B). Other parameter values are fixed as follows: d = 75, c = 7.5, m = 0.5, f = 0 and r = 10− 6. At the lower mutation rate, population growth rate increases the probability of the βS-like pattern when population size is small (Ne = 25,000) but decreases it when population size is large (Ne = 125,000). At a higher mutation rate, population growth has a consistent negative effect on the probability of the βS-like pattern. [file mmc2.pdf]

**$\beta^0$ -thalassaemia**

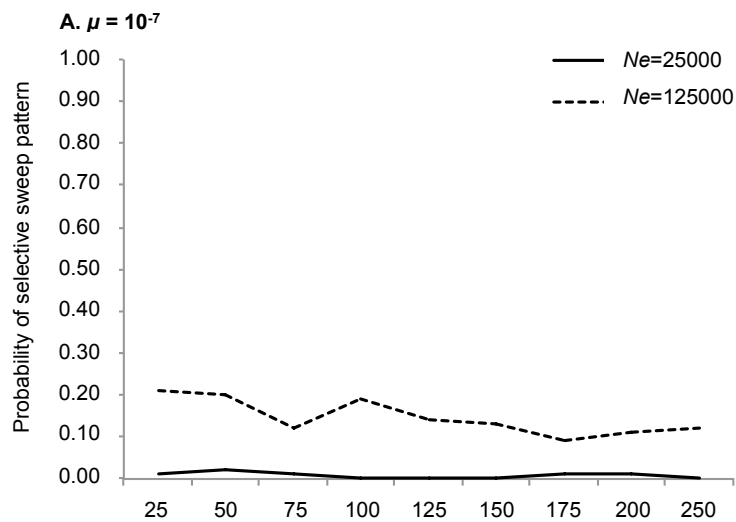

**$\beta^s$**

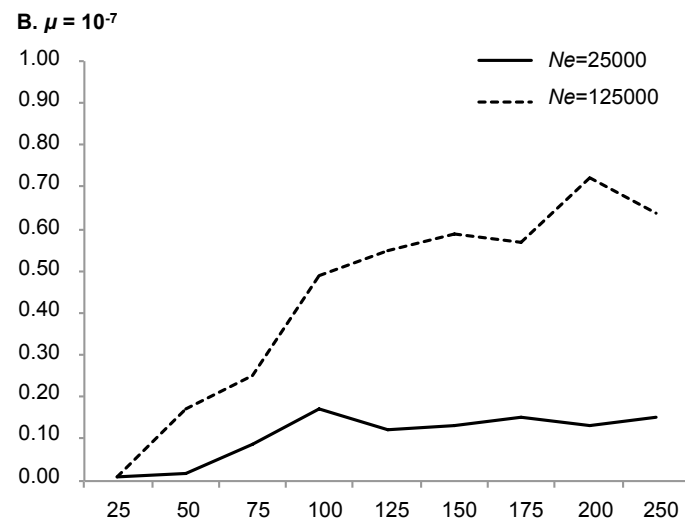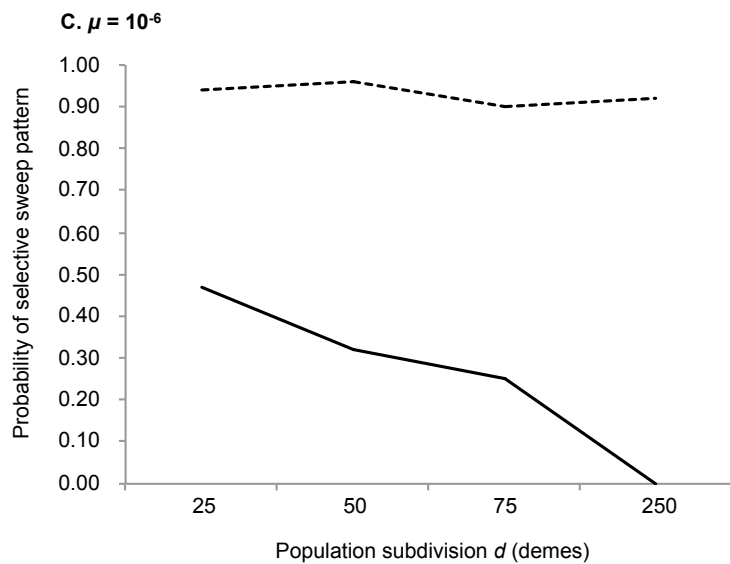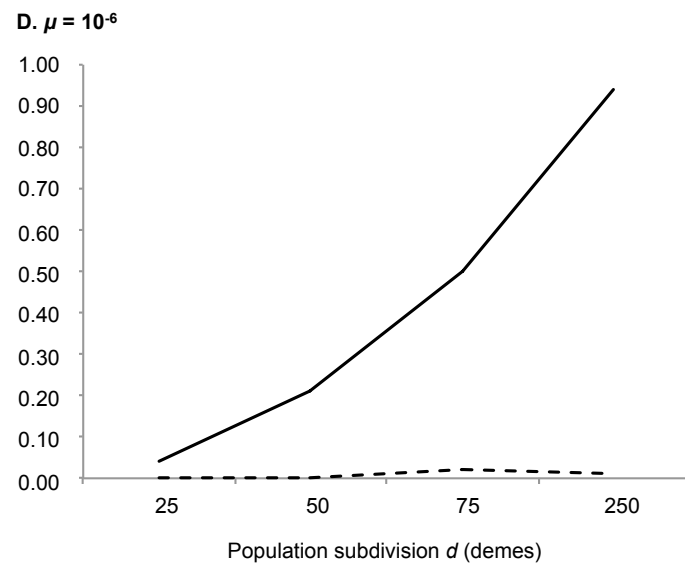

Supplement: Fig. S2 — The interaction between the effects of population subdivision and initial total population size on selective sweep outcomes. Each graph indicates how the probability of observing a β0-thalassaemia-like (A,C) or βS-like (B,D) pattern changes with different levels of population subdivision (x-axis). Each data point is based on 100 simulations. Results are shown for two different initial population sizes: Ne = 25000 (solid line) and Ne = 125000 (dashed line). Two different mutation rates are also shown: μ = 10− 7 (A,B) and μ = 10− 6 (C,D). In panels (A) and (C), m = 2, to maximise the probability of a β0-thalassaemia-like pattern; in panels (B) and (D) m = 0.5, to maximise the probability of a βS-like pattern. Other parameter values are fixed as follows: g = 0, c = 7.5, f = 0 and r = 10− 6. When both population size and mutation rate are low, opportunities for new copies of the allele to arise are few and therefore the speed at which alleles move through the network is less important in determining the patterns that emerges. By contrast, when both population size and mutation rate are high, so many haplotypes are generated that the β0-thalassaemia-like pattern is guaranteed whilst the βS-like pattern is precluded; regardless of how easy or difficult it is for alleles to move through the network. [file mmc3.pdf]

### $\beta^0$ -thalassaemia

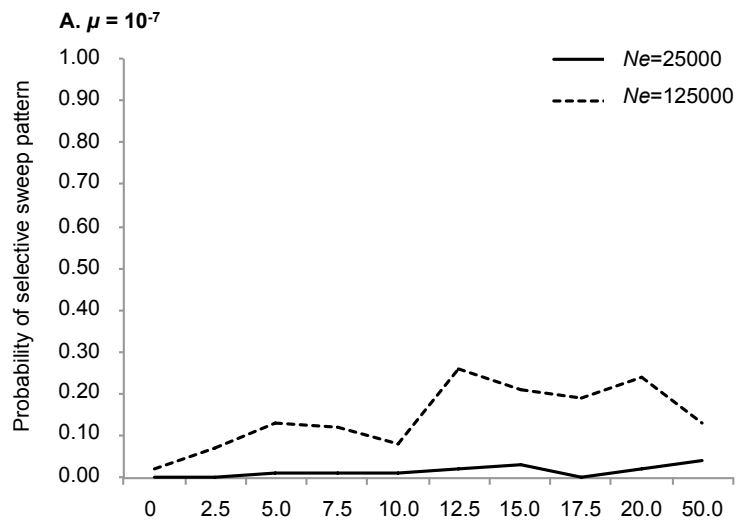

### $\beta^S$

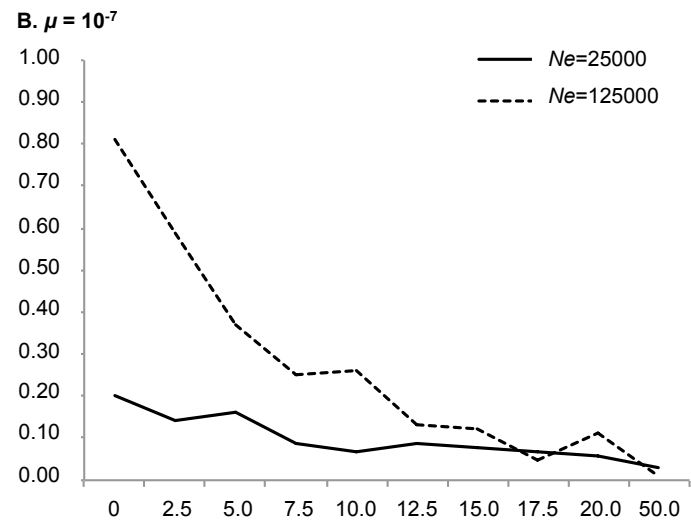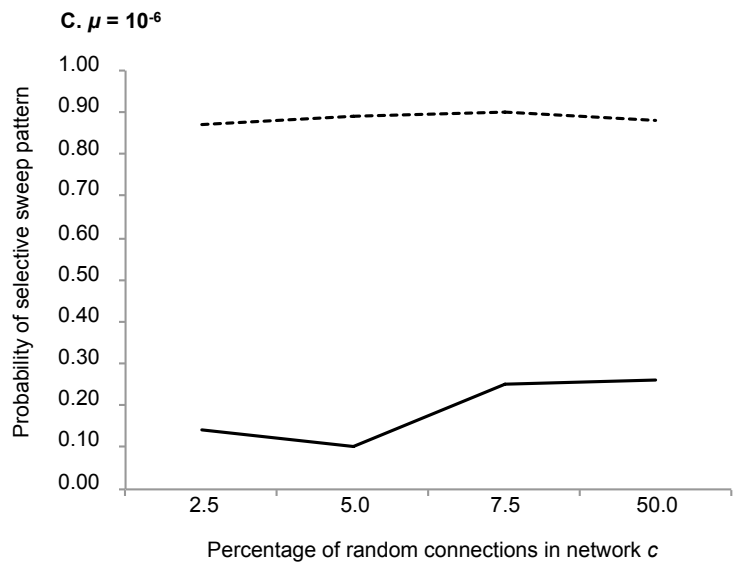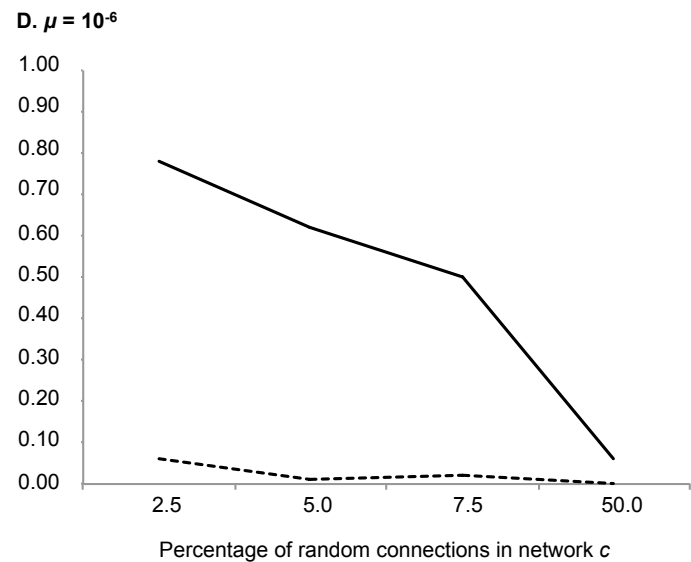

Supplement: Fig. S3 — The interaction between the effects of network connection structure and initial total population size on selective sweep outcomes. Each graph indicates how the probability of observing a β0-thalassaemia-like (A,C) or βS-like (B,D) pattern changes with different degrees of randomness in the migration network connection structure (x-axis). Each data point is based on 100 simulations. Results are shown for two different initial population sizes: Ne = 25,000 (solid line), and Ne = 125,000 (dashed line). Two different mutation rates are also shown: μ = 10− 7 (a,b) and μ = 10− 6 (c,d). In panels (A) and (C), m = 2, to maximise the probability of a β0-thalassaemia-like pattern; in panels (B) and (D) m = 0.5, to maximise the probability of a βS-like pattern. Other parameter values are fixed as follows: g = 0, d = 75, f = 0 and r = 10− 6. As for Supplementary Fig. S2, when both population size and mutation rate are low, opportunities for new copies of the allele to arise are few, and therefore the speed at which alleles move through the network is less important in determining the patterns that emerges. By contrast, when both population size and mutation rate are high, so many haplotypes are generated that that the β0-thalassaemia-like pattern is guaranteed whilst the βS-like pattern is precluded, regardless of how easy or difficult it is for alleles to move through the network. The interaction between the effects of network connection structure and initial total population size on selective sweep outcomes. Each graph indicates how the probability of observing a β0-thalassaemia-like (A,C) or βS-like (B,D) pattern changes with different degrees of randomness in the migration network connection structure (x-axis). Each data point is based on 100 simulations. Results are shown for two different initial population sizes: Ne = 25,000 (solid line), and Ne = 125,000 (dashed line). Two different mutation rates are also shown: μ = 10− 7 (a,b) and μ = 10− 6 (c,d). In panels (A) and (C [file mmc4.pdf]
